# Cow manure as a lignocellulosic substrate for fungal cellulase expression and bioethanol production

**DOI:** 10.1186/s13568-018-0720-2

**Published:** 2018-11-29

**Authors:** Qin Yan, Xinli Liu, Yanan Wang, Hongxing Li, Zhigang Li, Lin Zhou, Yinbo Qu, Zhonghai Li, Xiaoming Bao

**Affiliations:** 1 State Key Laboratory of Biobased Material and Green Papermaking, Qilu University of Technology, Shandong Academy of Sciences, Jinan, 250353 China; 2 Shandong Provincial Key Laboratory of Microbial Engineering, Department of Bioengineering, Qi Lu University of Technology, Shandong Academy of Sciences, Jinan, 250353 China; 3 State Key Laboratory of Microbial Technology, Shandong University, Jinan, 250100 China

**Keywords:** Cow manure, Bioethanol, Cellulase, *Penicillium oxalicum*

## Abstract

Conversion of various lignocellulosic materials into bioethanol is growing in demand but greatly depends on feedstock availability. Dairy cow manure is an agricultural waste widely distributed worldwide. This study investigated the induction of cellulases by cow manure and the conversion of cow manure materials into lignocellulosic ethanol. Alkaline NaOH pretreatment improved the accessibility of cow manure lignocellulose to enzymes followed by enzymatic hydrolysis using *Penicillium oxalicum* cellulases. The ethanol yields from pretreated cow manure and anaerobically digested cow manure were 0.19 and 0.13 g/g-raw biomass, respectively, using recombinant *Saccharomyces cerevisiae* strain LF1 designed for lignocellulosic ethanol production through simultaneous saccharification and fermentation. Fed-batch supplementation with cellulolytic enzymes and substrates after initial enzymatic hydrolysis also contributed to ethanol production up to 25.65 g/L. These results demonstrate that cow manure is a potential feedstock for inducing fungal cellulase expression and converting lignocellulose into bioethanol.

## Introduction

The production of first-generation biofuels from sources such as starch and vegetable oil has risen steeply over the last few years but compete with food crops (Martin 2010). Lignocellulosic biomass is the most abundant and attractive renewable energy resource in nature, and it is a valuable alternative to chemical feedstock and liquid transport fuels derived from petroleum (Ho et al. 2014). At present, lignocellulosic biomass used for bioethanol production is mainly derived from agricultural residues, agricultural wastes, energy crops, and forestry residues (Ho et al. 2014). Some lignocellulosic plant materials, such as softwoods, sugar cane crop, corn stover, and wheat straw, received attention for their potential conversion into biofuel (Himmel and Bayer 2009; Schubert 2006). The average glucan and xylan contents of these lignocellulosic biomass materials are about 65% of the overall lignocellulosic biomass composition based on dry weight (Mabee et al. 2006). These biomass materials need to undergo pretreatment to decrease the natural recalcitrance of lignocellulose, followed by enzymatic hydrolysis to convert them into sugars, and fermentation to produce second-generation biofuels (Taherzadeh and Karimi 2008). Until now, second-generation biofuels are still associated with immature commercial markets, and some technologies remain underdeveloped. Many of these problems could be addressed by fully utilizing lignocellulosic biomass feedstocks.

Currently, the utilization of lignocellulosic biomass feedstocks is mainly restricted to forestry residues, agricultural residues, and energy crops. For example, corn stover as a specific model biomass could theoretically produce the maximum amount of 0.23 g ethanol per gram raw biomass based on its average cellulose content (Johnson et al. 2016). Other agricultural wastes could also be used as sources of potential lignocellulosic biomass feedstocks for biofuels. Cow manure is the most widely distributed agricultural waste and is a typical lignocellulosic material (Ashekuzzaman and Poulsen 2011). For instance, a cow weighing 250 kg could excrete more than ten tons of manure per year on a dairy farm. According to the USDA cattle report, the average number of milk cows was 9.4 million head in the United States in 2017 (https://www.ers.usda.gov/webdocs/publications/87428/ldp-m284.pdf?v=43145), and the number of cows estimated in other nations was more than 150 million head. Thus, cows could produce large amounts of manure, which is a potential bioenergy source.

Traditionally, livestock manure was mainly directly injected into soil as fertilizer due to its abundant macronutrients for plant growth (Powell and Rotz 2015). At present, this waste is used to generate methane-rich biogas in oxygen-depleted environments (Ashekuzzaman and Poulsen 2011). This clean-burning biogas is produced by methanogenic bacteria from lignocellulosic substrates in manure to generate electricity and heat energy (Maranon et al. 2011). In recent years, biogas energy projects in countries such as China and France have been installed and used for reaping energy from manure on dairy farms (Loyon 2017; Yang et al. 2016). However, the climate and air pollution of biogas are important factors influencing the efficiency of continuous gas production from biogas digesters (Seppala et al. 2013). In addition, the potential of using cow manure has so far been unexploited, and only a limited lignocellulosic fraction in this green supply has been converted into biogas by microorganisms, with large amounts of anaerobically digested cow manure generated during the process (Diaz et al. 2016). The treated lignocellulosic fraction could be effectively disrupted by enzymatic hydrolysis into fermentable sugars (Zhao et al. 2011). From a carbon cycle perspective, it is an alternative to biogas for the production of bioethanol and other chemicals with lignocellulosic fraction in cow manure.

Fungal cellulases play a significant role in the enzymatic saccharification of pretreated biomass (van den Brink and de Vries 2011). These growing large-scale bioconversions of lignocellulosic materials have led to an increasing concern about the commercial production of cellulase (Cherry and Merino 2007). The cellulase activity produced by fungi is mainly composed of cellobiohydrolases, endoglucanases, β-glucosidases, and some auxiliary enzymes, and the hyper-expression of these proteins is still inducer dependent (Glass et al. 2013). Cellobiose and sophorose are the most effective but expensive inducers for commercial enzyme production (Bischof et al. 2016; Xu et al. 2014). Therefore, the present study investigated the feasibility of using cow manure for cellulase expression.

Presently, there is no available test to use cow manure as feedstock for fungal cellulase expression and bioethanol production. This paper described the conversion of cow manure and anaerobically digested cow manure into a cellulosic ethanol through pretreatment and efficient enzymatic hydrolysis. Furthermore, this study determined the most common composition of cow manure and anaerobically digested cow manure, whether cow manure and anaerobically digested cow manure have higher inducibility than delignined corn cob residue for cellulase expression in cellulolytic filamentous fungi, and whether the pretreated cow manure and anaerobically digested cow manure could be effectively disrupted by enzymatic hydrolysis into fermentable sugars and have high bioethanol conversion yield. Taken together, the research has implications for all dairy farms as it is the first attempt to investigate the potential utilization of cow manure for lignocellulosic-ethanol in combination with lignocellulolytic enzyme production, which could serve as a reference for improving agricultural waste economics.

## Materials and methods

### Sample collection, strains, and growth conditions

Cow manure and anaerobically digested cow manure were collected from a dairy farm (Shandong Province, China). The collected fresh cow manure slurry was then introduced into continuous digesters for methane generation. The anaerobic digestion could efficiently occur between 25 °C and 40 °C. In this process, organic substances are decomposed and converted into biogas by methane-producing bacteria under anaerobic conditions. *Saccharomyces cerevisiae* LF1 (Li et al. 2016) was cultured in YPD medium (1% yeast extract, 2% peptone, and 2% glucose) at 30 °C and 200 rpm. To induce cellulase expression, we precultivated *Penicillium oxalicum* C1-9 (Yao et al. 2016) on Vogel’s medium containing 1 × Vogel’s salt solution and 2% glucose for 26 h. A 50 × Vogel’s salt solution stock containing the following was prepared: 125 g/L Na_3_Citrate·2H_2_O, 250 g/L KH_2_PO_4_, 100 g/L NH_4_NO_3_, 10 g/L MgSO_4_·7H_2_O, 5 g/L CaCl_2_·2H_2_O, 0.25 mg/L biotin, and 5 mL/L trace element solution (50 g/L Citric acid·H_2_O, 50 g/L ZnSO_4_·7H_2_O, 10 g/L Fe(NH_4_)_2_(SO_4_)_2_·6H_2_O, 2.5 g/L CuSO_4_·5H_2_O, 0.5 g/L MnSO_4_·1H_2_O, 0.5 g/L H_3_BO_3_, and 0.5 g/L Na_2_MoO_4_·2H_2_O). The vegetative mycelia were collected through vacuum drum filtration and then inoculated into 500 mL flasks containing 100 mL of fermentation medium at an initial pH of 5.5 at 30 °C and 200 rpm. Fermentation medium contained 0.60% Avicel, 2% corn cob residue, 4.66% wheat bran, 1% soybean cake power, 0.20%(NH_4_)_2_SO_4_, 0.10% urea, 0.28% NaNO_3_, 0.30% KH_2_PO_3_, and 0.05% MgSO_4_·7H_2_O. To test cellulase production by *P. oxalicum* C1-9 using cow manure, we added 4% cow manure or 4% anaerobically digested cow manure to replace 2% corn cob residue in fermentation medium.

### Enzyme assays

The filter paper enzyme (FPase) and endoglucanase (CMCase) activities of the culture supernatants were assayed using the DNS method (20.8 g/L sodium hydroxide, 6 g/L 3,5-dinitrosalicylic acid, 6 g/L potassium sodium tartrate, 5 g/L sodium sulfite anhydrous, and 5 g/L redistilled phenol) against Whatman No.1 filter paper and carboxymethyl cellulose sodium salt (CMC-Na), respectively. Crude enzyme samples were centrifuged at 12,000 rpm (4 °C, 10 min), and the supernatants were transferred into a new centrifuge tube and placed on ice until used in enzyme assays. A 50 mg Whatman No. 1 filter paper for FPase was added to 2 mL of crude enzyme solution (diluted to the appropriate range using HAC-NaAC buffer, pH 4.8), and a 1.5 mL volume of CMC-Na (1%, m/v) for CMCase was added to 0.5 mL of crude enzyme solution (diluted to the appropriate concentration using HAC-NaAC buffer, pH 4.8). The resulting reaction mixtures were mixed well and incubated at 50 °C for 1 h for FPase or at 50 °C for 30 min for CMCase. Then, a 3 mL volume of DNS was added to terminate the reactions. The reaction mixtures were subsequently placed in boiling water for 10 min. Distilled water was added to maintain a constant volume of 25 mL as the liquid cooled. The optical densities (ODs) of the reaction solutions were determined using a microplate reader at a wavelength of 540 nm. The activities of *p*NPCase, *p*NPXase, and *p*NPGase were measured by using *p*-nitrophenyl-β-d-cellobioside (*p*NPC), *p*-nitrophenyl-β-d-xylopyranoside (*p*NPX), or *p*-nitrophenyl-β-d-glucopyranoside (*p*NPG) as substrates, respectively. A 150 µL reaction mixture contained 50 µL of *p*NPC/*p*NPX/*p*NPG (1 mg/mL) and 100 µL of diluted crude enzyme solution using NaAC buffer. The mixture was mixed well and incubated at 50 °C for 30 min. Then, the reaction was terminated using a 150 µL volume of 10% Na_2_CO_3_ (w/v) solution. The released *p*-nitrophenol was measured by assessing the OD at a wavelength of 420 nm. The above substrates were dissolved in NaAC buffer (50 mM, pH 4.8), and the *p*NPC solution additionally contained 10 mg/mL d-glucono-δ-lactone. Enzyme activity units for all enzymes were defined as the amount of enzyme required to produce 1 µmol of product (glucose or *p*-nitrophenol) per minute under experimental conditions.

Stacking and separation gel of SDS-PAGE analysis was prepared by 12.5% Omni-PAGE™ Precast Page Gel Fast DIY Kit. The mixture of 20 µL of crude enzyme solution and 5 µL of 5 × Loading buffer was boiled for 10 min and then added to the gel. SDS-PAGE analysis was performed in glycine buffer with 80 V for stacking gel and 120 V for separation. For staining, the gel was placed in 10% acetic acid in water containing 60 mg/L Coomassie Brilliant Blue R-250 for 2 h. For destaining, the gel was placed in 10% acetic acid (glacial acetic acid: ethanol: distilled water, 1: 1: 8, v/v/v) for 2–24 h.

### Pretreatment and component analysis

Before material pretreatment, cow manure and anaerobically digested cow manure samples were briefly milled using a micromiller and then completely dried at 45 °C. All the materials were weighed and divided into two groups: one group used for component analysis and the other for pretreatment. Pretreatment was carried out in a conical flask of 500 mL with a ratio of 1 g sample per 10 mL of 2% sodium hydroxide (m/v) or 2% sulfuric acid. Pretreatment was performed at 121 °C for 30 min. After the pretreatment, biomasses were cooled and washed using running tap water (130 mL/g) until a neutral pH level for the samples was achieved. The pretreated materials were again dried at 45 °C. The dried materials were weighed and stored in plastic bags at 4 °C until use.

Compositional analysis of lignocellulosic materials was performed using a modified version of established NREL (US Department of Energy, 2006) protocols. Untreated and pretreated biomass samples were pre-hydrolyzed with 12 mol/L sulfuric acid (72%, w/w) at 30 °C for 2 h and then mixed thoroughly every 10 min. The samples were diluted with water to a final sulfuric acid concentration of 0.41 mol/L and then hydrolyzed at 121 °C for 1 h. Following hydrolysis, 1 mL of the hydrolysate supernatant was taken, pH adjusted to 1–3 with Ba(OH)_2_, and then centrifuged at 12,000 rpm (4 °C, 10 min). The resulting supernatant was filtered through a 0.22 µm microporous membrane to remove impurities. Filtered samples were stored at 4 °C before HPLC analysis. The remaining residue after hydrolysis was collected by vacuum filtration, and rinsed with distilled water, and then dried at 105 °C to constant weight for determination of lignin content.

### Simultaneous saccharification and fermentation (SSF)

The pretreated lignocellulosic materials at high solids loading (10% or 15%, w/v) were pre-hydrolyzed into a liquid hydrolysate slurry using Cellulases SN-1 and C1-9 at a dosage of 20 FPU per gram of dry biomass or complex cellulases (commercial cellulase SN-1 10 FPU/g and C1-9 enzyme 10 FPU/g), respectively. HAC-NaAC buffer (50 mM, pH 4.8) was added in a final volume of 20 mL. After the cellulose and hemicellulose were enzymatically hydrolyzed into sugars such as glucose and xylose, we added 5‰ activated *S. cerevisiae* LF1 (5 mg *S. cerevisiae* LF1 per 1 g of biomass) into the hydrolysates to proceed for 72 h at 30 °C and 200 rpm. *S. cerevisiae* LF1 uses glucose and xylose under anaerobic respiration conditions to produce ethanol. Fermentation broth samples were withdrawn at different time points of 0, 24, 48, and 72 h, respectively. All flask SSF fermentation experiments were performed in triplicate.

High solid loading of feedstock might directly lead to high ethanol titer. Fed-batch hydrolysis experiments were tested. The initial hydrolysis was performed using substrate concentration 10% (m/v) with 20 FPU/g cellulase SN-1 in a short period (12 h) at 50 °C, pH 4.8. Additional 5% or 10% (w/v) of the pretreatment material and *S. cerevisiae* LF1 were added into the initial enzymatic hydrolysis complex.

### Analytical methods

Fermentation broth was centrifuged at 4 °C, 12,000 rpm. The supernatant was transferred to a new 1.5 mL centrifuge tube. This tube was placed in a boiling water bath for 5 min and then centrifuged again as above. The resultant supernatants were filtered through 0.22 µm organic filters, and the filtered samples were stored at 4 °C prior to HPLC analysis.

Glucose, xylose, cellobiose, and ethanol were analyzed on HPLC equipped with a refractive index detector and a Carbomix H-NP10 column. A 2.5 mM H_2_SO_4_ solution was used as flow phase at a flow rate of 0.6 mL/min at 55 °C. The following formula was used to calculate ethanol conversion efficiency (ECE):

$$ECE = {\raise0.7ex\hbox{${c \times v}$} \!\mathord{\left/ {\vphantom {{c \times v} {(mz - mc) \times 0.511}}}\right.\kern-0pt} \!\lower0.7ex\hbox{${(mz - mc) \times 0.511}$}} \times 100\% ,$$

where *c* represents the concentration (g/L) of ethanol in the SSF measured by HPLC analysis, *v* represents the total volume (L) of fermentation broth, *m*_*z*_ represents the total amount of lignocellulose and hemicellulose components in the biomass that are completely hydrolyzed to monosaccharide (g), and *m*_*C*_ represents the mass of residual sugar in the fermentation broth and residual solid after co-fermentation (g). The 0.511 represents the conversion factor of glucose and xylose to ethanol.

## Results

### Composition analysis and pretreatment of cow manure

**Fig. 1:**
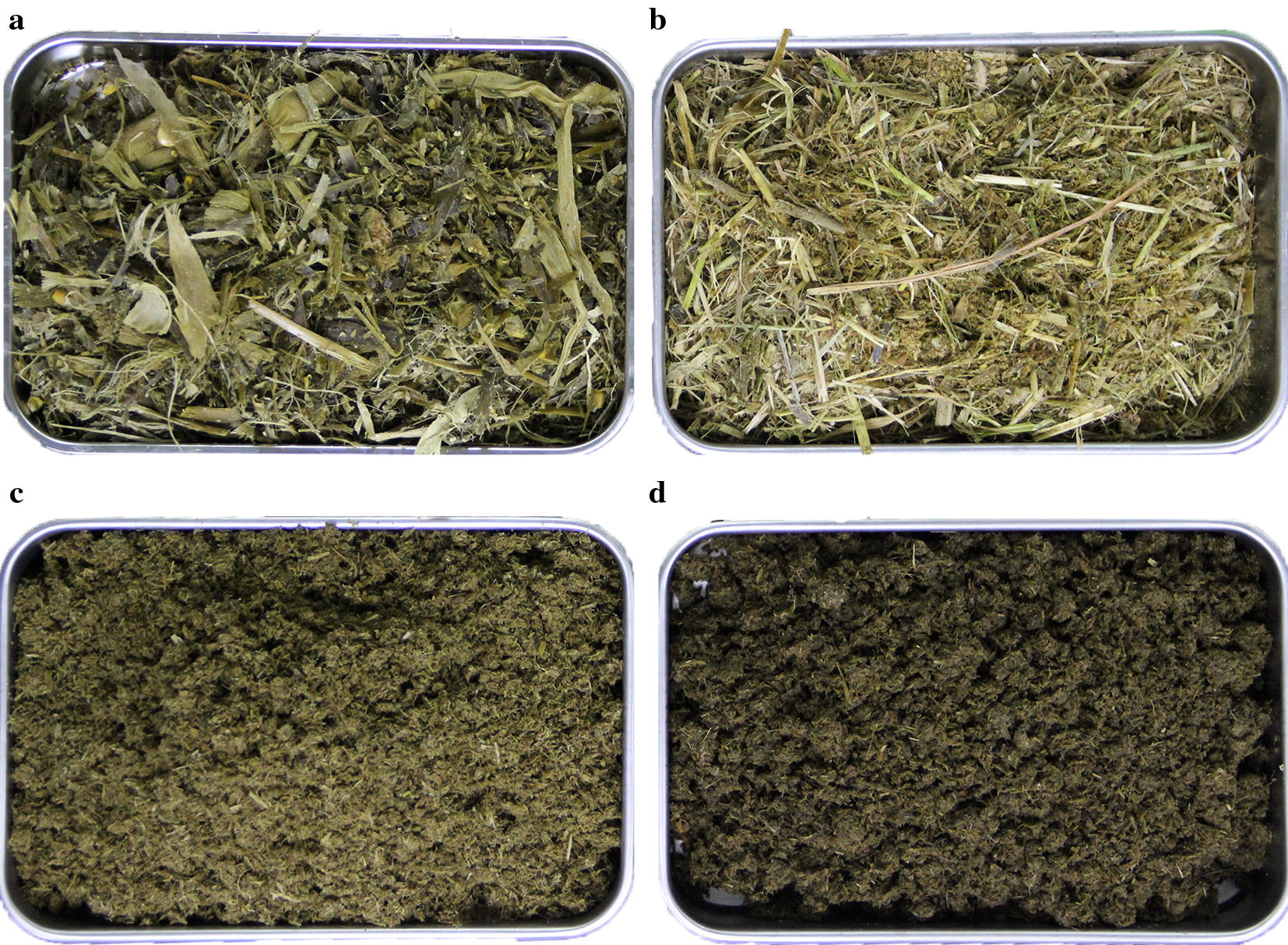
Images for corn silage diets and cow manure and anaerobically digested cow manure. Corn silage diets (**a**), the coarse milled corn silage diets supplemented with additional protein feed (**b**), solid cow manure materials after removing the liquid part by mechanical packing (**c**), anaerobically digested cow manure obtained from physical separation of slurry after anaerobic digestion process for biogas production (**d**)

Many previous studies focused on lignocellulosic feedstock for bioethanol production (Ho et al. 2014). Among these lignocellulosic materials, agriculture wastes with high carbohydrate content are promising candidates of biodegradable sources due to their potential commercial application prospects in bioenergy (Balan 2014; Haq and Easterly 2006; Zhao et al. 2011). Cows are fed with corn silage (Fig. 1a, b) with a high proportion of lignocellulosic fiber in dairy diets and produce manure after digestion (Hassanat et al. 2017). Therefore, cow manure (Fig. 1c) is one of the most common and readily available agriculture waste on factory farms. At present, cow manure is mainly used to generate methane-rich biogas and fertilizer due to its abundant macronutrients for the growth of microorganisms and plants. To develop a feasible enzymatic production and cellulosic ethanol conversion process using solid cow manure as feedstock, we carefully investigated the lignocellulose compositions in cow manure and anaerobically digested cow manure (Fig. 1d, solid residue after biogas generation). The composition results for cow manure are shown in Table 1. The results showed that cow manure was composed of glucan (16.62%) and xylan (15.26%). The content of lignin in cow manure was (50.59%). Feedstock seasonality and different feedstock type might slightly impact the overall conversion process design (Van Dyk and Pletschke 2012). The glucan content remaining in the anaerobically digested cow manure after biogas production relatively decreased compared with that in the unfermented cow manure because of the degradation of the lignocellulose faction during biogas production within the anaerobic reactor. Thus, anaerobically digested cow manure contained glucan (14.50%) and xylan (12.56%). These data revealed that cow manure contained relatively high lignocellulosic content and that the anaerobic digestion of cow manure for methane fermentation slightly decreased the proportion of lignocellulosic fiber.

**Table 1:** Composition of cow manure and anaerobically digested cow manure

Biomas components	Cellulose (%)	Hemicellulose (%)
Cow manure	16.62	15.26
Alkali treated cow manure	35.34	15.48
Acid treated cow manure	26.62	7.61
Anaerobically digested cow manure	14.50	12.56
Alkali treated anaerobically digested cow manure	28.94	15.98
Acid treated anaerobically digested cow manure	22.56	3.22

Cellulose accessibility to cellulolytic enzymes is limited by complex lignin carbohydrate bonds (Vermaas et al. 2015). Therefore, lignocellulosic material pretreatment is the first step to efficiently produce fermentable sugars before cellulosic ethanol production from the lignocellulosic biomass of cow manure (Taherzadeh and Karimi 2008). Physical treatment was performed to increase the surface area of air-dried cow manure materials by milling, followed by incubation of cow manure with alkaline NaOH solution to disrupt the recalcitrant structure. Then, an additional detoxification step by washing with water was performed to remove additional inhibitory compounds to microbial fermentation. As shown in Table 1, pretreatment with 2% NaOH resulted in 35.34% cellulose and 15.48% hemicellulose in the pretreated cow manure and 28.94% cellulose and 15.98% hemicellulose in the pretreated anaerobically digested cow manure.

Dilute-acid pretreatment disrupts the structure of biomass materials and further removes lignin and hemicelluloses, which promotes the enzymatic hydrolysis to cellulose (Taherzadeh and Karimi 2008). To investigate the effect of dilute-acid pretreatment on the hydrolysis of the cow manure, we incubated cow manure with 2% sulfuric acid solution. Hemicellulose in dilute-acid pretreated cow manure could exhibit higher degradation. The effects of pretreatment on cow manure and anaerobically digested cow manure were analyzed based on the sugar production during pretreatment. As shown in Table 1, dilute-acid pretreatment resulted in 26.62% cellulose and 7.61% hemicelluloses in the pretreated cow manure and 22.56% cellulose and 3.22% hemicelluloses in the pretreated anaerobically digested cow manure. Compared with 2% NaOH pretreatment, pretreatment of cow manure using 2% sulfuric acid solution resulted in low contents of glucose and xylose in solid residues. Therefore, alkali treatment was generally more effective in the pretreatment of cow manure and anaerobically digested cow manure in this study.

### Activity comparison of delignined corn cob residue and cow manure-based cellulase

**Fig. 2:**
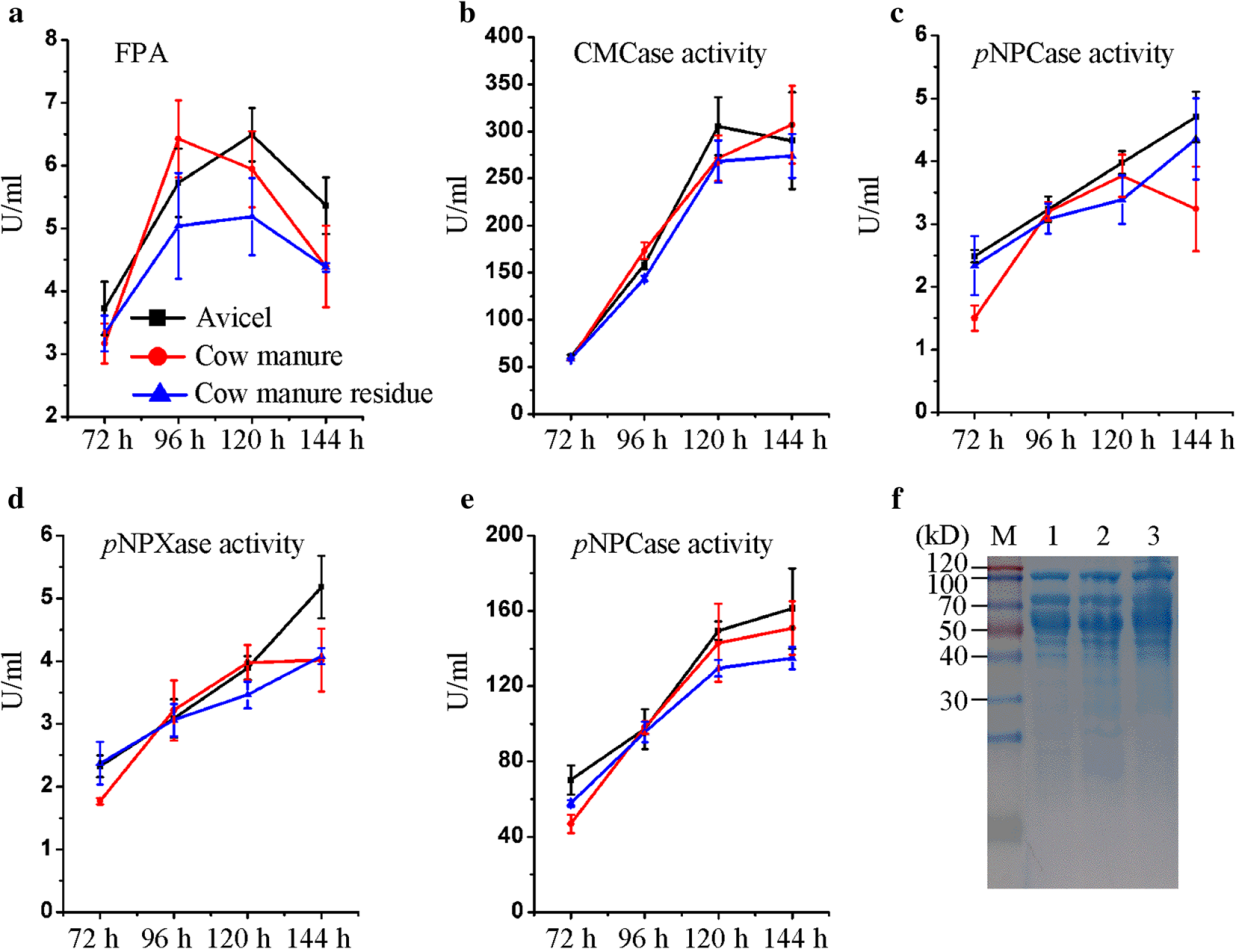
Time course of cellulolytic enzyme activities by *P. oxalicum*. **a**–**e** Under original production medium (solid squares), cow manure (solid circles), anaerobically digested cow manure (triangles). Data presented are average of triplicate experiments; error bars indicate the standard deviations. **f** SDS-PAGE of proteins from unconcentrated culture supernatants of *P. oxalicum* C1-9 when cultured in cow manure medium (lane 2) and anaerobically digested cow manure medium (lane 3) as compared original production medium (lane 1) inducing cellulase expression for 96 h

Enzymatic conversion of pretreated lignocellulose materials into monosaccharides such as glucose and xylose is needed during lignocellulosic ethanol process, where lignocellulolytic enzymes are used to catalyze the hydrolysis of lignocellulosic substrates (Cherry and Merino 2007). Previous studies have confirmed that the cost of cellulase production affects the cost estimates for cellulosic ethanol process, especially with the off-site approach for producing cellulase (Cherry and Merino 2007). Then, lowering the cellulase production cost on-site by integrated production is needed to render lignocellulosic ethanol economically viable (Johnson et al. 2016). To compare the performances and assess whether the cow manure could effectively induce the cellulase expression, we used cow manure as the primary substrate to substitute a primary feedstock delignined corn cob residue (from xylitol production) in the original production medium inducing cellulase expression (Han et al. 2016). *P. oxalicum* strain C1-9 has been proposed for use in the cellulase production because it is a recombinant strain engineered from wild-type strain 114-2 after tertiary genetic recombination (Yao et al. 2016). In addition, *P. oxalicum* strain C1-9 has high cellulase-producing ability under cellulose-inducing conditions (Yao et al. 2016). In this study, when grown on delignined corn cob residue as primary feedstock (Fig. 2), *P. oxalicum* strain C1-9 showed maximal activities for filter paper enzyme activity (FPA, 6.49 ± 0.43 U/mL), cellobiohydrolase (pNPCase, 4.70 ± 0.40 U/mL), endoglucanase (CMCase, 305.36 ± 30.68 U/mL), β-glucosidase (β-BGase, 161.28 ± 21.25 U/mL), and β-xylosidase (BXase, 5.18 ± 0.50 U/mL). When grown on cow manure as primary feedstock, *P. oxalicum* strain C1-9 showed maximal activities for FPA (6.42 ± 0.61 U/mL), pNPCase (3.77 ± 0.33 U/mL), CMCase (307.06 ± 41.31 U/mL), β-BGase (150.88 ± 14.25 U/mL), and BXase (4.01 ± 0.50 U/mL). The volume of FPA and the protein expression patterns in the supernatant on cow manure were comparable to those on the original production medium (Fig. 2). These data signified that cow manure could be efficiently hydrolyzed and might have comparable inducibility for cellulase expression to delignined corn cob residue by substitution of primary feedstock on cellulase-producing medium.

The cost of cellulase production is significant during enzymatic hydrolysis (Johnson et al. 2016). Therefore, reducing this cost is important to render cellulosic ethanol viable. Integrating cellulase and bioethanol production by using the same feedstock could lower the cost of cellulase production, which might be a better choice over traditional off-site cellulase production without the use of stabilizers and cellulase transport (Johnson et al. 2016). In the present study, cow manure as the primary feedstock was used to produce cellulase. The results showed that cow manure could be considered a promising inducer of cellulase expression with de-repressed strain and reduced cost of bioethanol production.

To further test whether anaerobically digested cow manure has similar cellulase expression inducibility, we used anaerobically digested cow manure as the primary substrate in cellulase-producing medium. As shown in Fig. 2, *P. oxalicum* strain C1-9 showed maximal activities for FPA (5.19 ± 0.61 U/mL), pNPCase (4.36 ± 0.65 U/mL), CMCase (273.93 ± 23.29 U/mL), β-BGase (134.96 ± 5.94 U/mL), and BXase (4.08 ± 0.12 U/mL). Although the strain C1-9 displayed slightly stronger pNPCase and BXase activities on anaerobically digested cow manure than on cow manure, its FPA, CMCase, and β-BGase activities declined more on anaerobically digested cow manure than on cow residue. These results demonstrated that the biogas production of cow manure reduced the glucan content in anaerobically digested cow manure and then significantly affected its inducibility in cellulolytic enzyme expression.

### Batch enzymatic hydrolysis and ethanol production

**Fig. 3:**
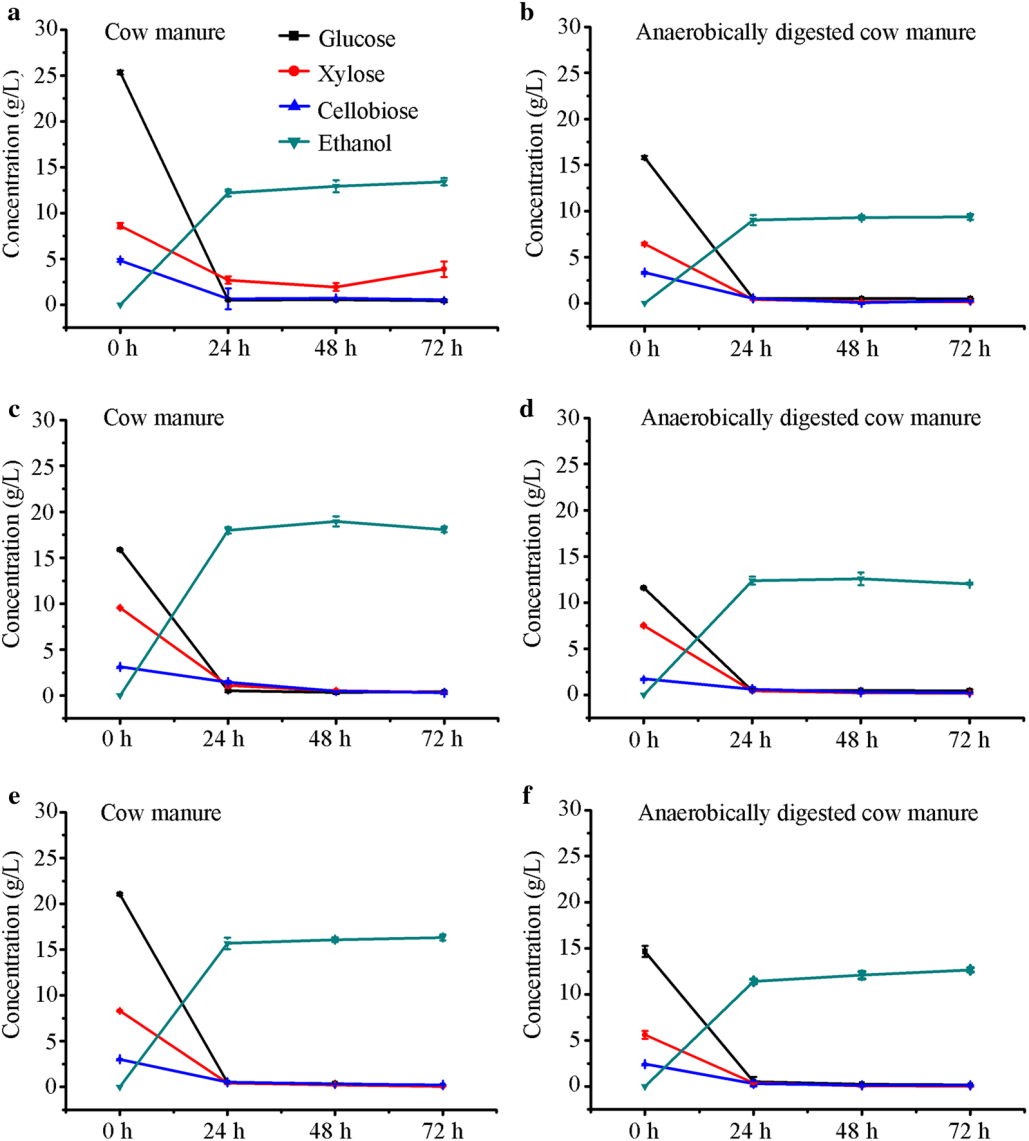
Batch enzymatic hydrolysis with commercial cellulase SN-1 and ethanol production. Hydrolysis of alkali treated substrates by C1-9 cellulase (**a**, **b**) was compared with commercial cellulase SN-1 (**c**, **d**) and combined hydrolysis by mixed enzymes (50% SN1 and 50% C1-9) at solid loading of 10% and enzyme loading 20 FPU/g dried substrates (**e**, **f**). Glucose (solid squares), xylose (solid circles), cellobiose (solid upward-facing triangles), ethanol (solid downward-facing triangles). Data presented are averages of triplicate experiments; error bars indicate the standard deviations

Enzymatic hydrolysis of biomass materials is critical for the viability of bioethanol production (Cherry and Merino 2007). Both of C1-9 complete enzyme and commercial cellulase SN-1 produced from *P. oxalicum* were used to perform comparable enzymatic hydrolysis. First, the cellulolytic activities for C1-9 complete enzyme and commercial cellulase SN-1 were determined, which showed the activities of FPA (70.86 U/mL), CMCase (1795.35 U/g), pNPCase (0.36 U/g), and BGase (730.37 U/g) and the activities of FPA (81.63 U/g), CMCase (2132.74 U/g), pNPCase (0.30 U/g), and BGase (18.64 U/g). Compared with SN-1, enzyme C1-9 exhibited much stronger BGase activities. When the pretreated substrate was saccharified using C1-9 enzyme at a load of 20 FPU/g dry substrate, the hydrolysates from 10% initial NaOH-pretreated cow manure showed 25.35 ± 0.21 g/L glucose, 8.63 ± 0.29 g/L xylose, and 4.83 ± 0.15 g/L cellobiose, and anaerobically digested cow manure hydrolysates showed 15.82 ± 0.18 g/L glucose, 6.43 ± 0.14 g/L xylose, and 3.33 ± 0.10 g/L cellobiose (Fig. 3a, b). Under commercial enzyme SN-1 hydrolysis conditions, 15.87 ± 0.09 g/L glucose, 9.5 ± 0.05 g/L xylose, and 3.12 ± 0.03 g/L cellobiose were achieved in the pretreated cow manure hydrolysates (Fig. 3c, d), whereas the anaerobically digested cow manure hydrolysates showed 11.60 ± 0.08 g/L glucose, 7.50 ± 0.09 g/L xylose, and 1.74 ± 0.01 g/L cellobiose (Fig. 3c, d). At a total complex enzyme load of 20 FPU/g dry substrate (SN-1 10 FPU/g and C1-9 10 FPU/g), 10% initial pretreated cow manure showed 21.05 ± 0.10 g/L glucose, 8.31 ± 0.06 g/L xylose, and 3.02 ± 0.03 g/L cellobiose, whereas the anaerobically digested cow manure hydrolysates showed 14.66 ± 0.60 g/L glucose, 5.60 ± 0.42 g/L xylose, and 2.42 ± 0.02 g/L cellobiose (Fig. 3e, f). This result indicated that high BGase activity in enzyme C1-9 (730.37 U/g) enhanced glucose content in the hydrolysates. In general, the production of glucose and cellobiose during enzymatic hydrolysis was higher with C1-9 lignocellulolytic enzymes than with commercial enzyme in either cow manure or anaerobically digested cow manure hydrolysates, and the enzymatic conversion of the pretreated cow manure to monomeric glucose and xylose demonstrated a potential to perform a lignocellulose-to-ethanol process.

The recombinant *S. cerevisiae* LF1 could efficiently use five- and six carbon sugars to ethanol fermentation (Li et al. 2016). Therefore, *S. cerevisiae* LF1 was proposed for the production of cellulosic ethanol from cow manure and anaerobically digested cow manure carbohydrates. Under batch mode by the SN-1 enzymatic hydrolysis of NaOH-pretreated cow manure and anaerobically digested cow manure, *S. cerevisiae* LF1 produced 18.94 ± 0.55 and 12.57 ± 0.70 g/L ethanol by co-fermentation of glucose and xylose from 10% initial solid load (Fig. 3c, d), respectively. The residual glucose and xylose concentrations were 0.41 and 0.87 g/L in the supernatant from the pretreated cow manure, respectively. However, when with lignocellulolytic enzyme C1-9, the ethanol yields from the pretreated cow manure and anaerobically digested cow manure were only 13.43 ± 0.39 and 9.37 ± 0.31 g/L ethanol (Fig. 3a, b), respectively. The residual glucose and xylose concentrations were 0.30 and 0.79 g/L in the supernatant from the pretreated cow manure, respectively. When using the complex enzyme from SN-1 and C1-9, the ethanol yield from the pretreated cow manure and anaerobically digested cow manure were 16.33 ± 0.33 and 12.65 ± 0.22 g/L ethanol (Fig. 3e, f), respectively. Therefore, the hydrolysates by enzyme C1-9 had higher initial sugar concentration but lower ethanol production than those by commercial enzyme SN-1.

**Fig. 4:**
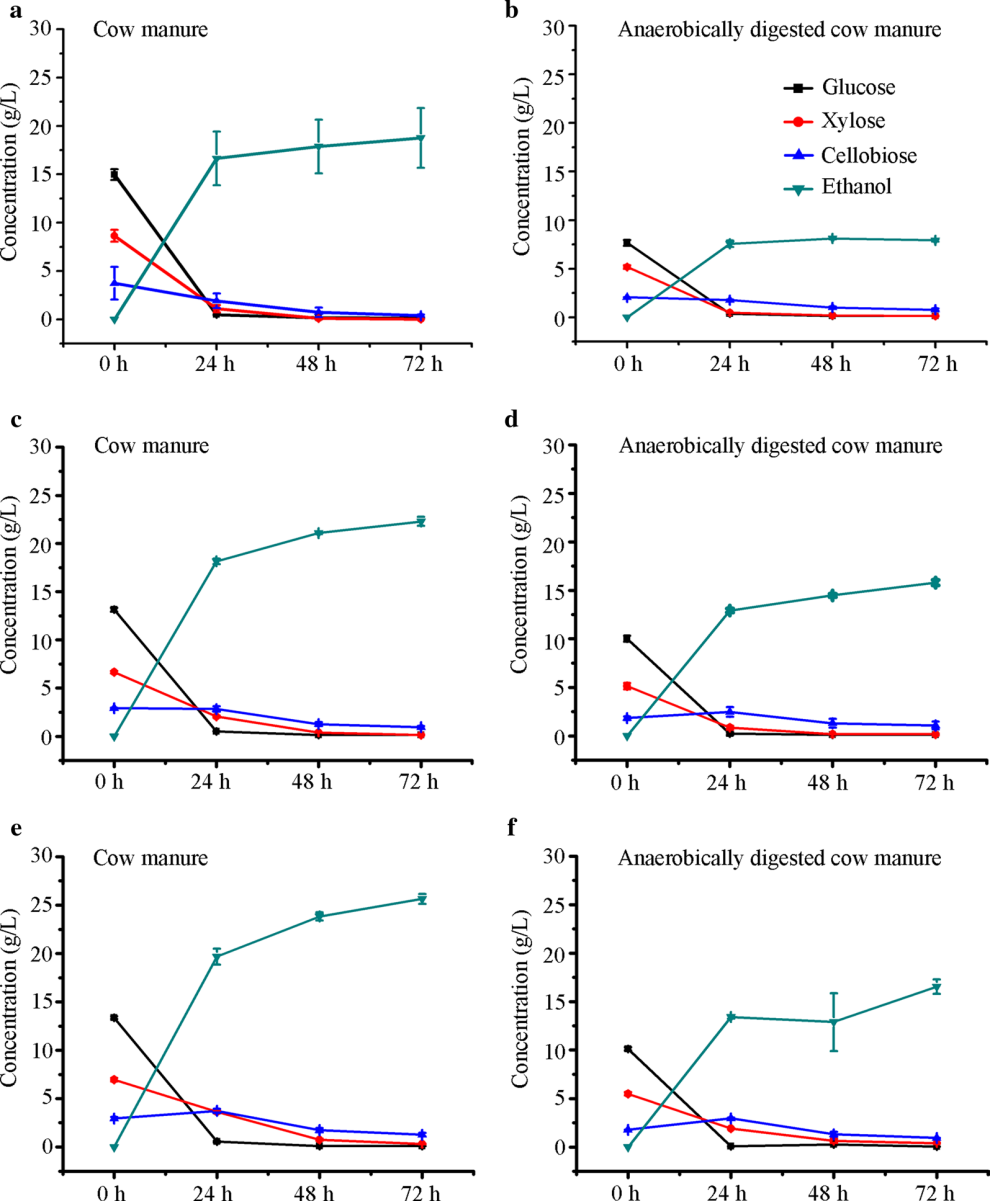
Batch and Fed-batch enzymatic hydrolysis with SN-1 and ethanol production. Batch enzymatic hydrolysis at an initial solid loading of 15% (**a**, **b**). Fed-batch enzymatic hydrolysis at an initial solid loading of 10%, and the final 15% (**c**, **d**) and 20% (**e**, **f**) biomass of fed-batch supplement were performed

The maximum glucan conversion rates of the NaOH-pretreated cow manure and anaerobically digested cow manure by *P. oxalicum* cellulases were 71.73% and 54.66% at 10% solid loading, respectively. To better advance the enzymatic hydrolysis of cow manure for biological conversion to ethanol, we further investigated bioethanol production using elevated solid loading. When enzymatic hydrolysis using commercial enzyme SN-1 was carried out at an enzyme load of 20 FPU/g dry substrate, the hydrolysate from the 15% initial NaOH-pretreated cow manure showed 14.97 ± 0.55 g/L glucose, 8.64 ± 0.61 g/L xylose, and 3.74 ± 1.68 g/L cellobiose, and the anaerobically digested cow manure hydrolysates showed 7.67 ± 0.30 g/L glucose, 5.17 ± 0.19 g/L xylose, and 2.06 ± 0.01 g/L cellobiose (Fig. 4a, b). Under batch mode, *S. cerevisiae* LF1 produced 18.76 ± 3.09 and 8.12 ± 0.09 g/L ethanol from 15% initial solid loading hydrolysates for cow manure and anaerobically digested cow manure, respectively (Fig. 4a, b). The sugar yields and ethanol production of 15% initial substrates were almost similar to those of 10% initial substrates across saccharification and ethanol fermentation. These results deviated from the predicted values and indicated that simply boosting initial solid load from 10% to 15% (m/v) did not improve glucan and xylan conversions and ethanol production.

### Fed-batch enzymatic hydrolysis and ethanol production

The initial slurry with feed material at elevated solid loading was not effectively flowing by gravity in the conical flask with a stopper. To overcome this problem in the above batch operation of 15% initial solid load, we performed fed-batch enzymatic hydrolysis to enhance the total fermentable sugar concentration. The glucose, xylose, and cellobiose concentrations were 13.15, 6.67, and 2.92 g/L at 10% initial substrate consistency after 12 h enzymatic hydrolysis (Fig. 4c, d). Then, fed-batch supplementation of cow manure (final 15% biomass, m/v) and SN-1 enzyme was implemented to enhance total sugar production, and 22.29 g/L ethanol was subsequently obtained by co-fermentation of glucose and xylose from the cow manure hydrolysates (Fig. 4c). The residual glucose and xylose concentrations were 0.53 and 1.16 g/L in the supernatant from the pretreated cow manure, respectively. Similarly, when anaerobically digested cow manure was hydrolyzed, the glucose, xylose, and cellobiose concentrations were 10.02, 5.13, and 1.83 g/L, respectively, and then 15.80 g/L ethanol was obtained (Fig. 4d). The controlled addition of the cow manure and lignocellulolytic enzymes by fed-batch enzymatic hydrolysis directly increased ethanol production. Thus, the yield of the desired product bioethanol might be further increased by increasing feed material concentration.

Therefore, the fed-batch enzymatic saccharification at elevated solid loading up to 20% (w/v) was further carried out. The initial enzymatic conversion of 10% pretreated cow manure was also performed at an enzyme load of 20 FPU/g substrate. After the initial 12-hour enzymatic saccharification, the sugar concentration values at 10% initial solid consistency were 13.37 g/L (glucose), 6.97 g/L (xylose), and 2.95 g/L (cellobiose), which were similar to the above results (Fig. 4e). Subsequently, fed-batch supplementation of the substrate and fermentation with *S. cerevisiae* were implemented. Then, 25.65 and 16.54 g/L ethanol were obtained from the pretreated cow manure and anaerobically digested cow manure, respectively (Fig. 4e, f). These results demonstrate that fed-batch enzymatic hydrolysis is an efficient procedure for enhancing ethanol production, and increasing the supplement of the pretreated cow manure and anaerobically digested cow manure could increase ethanol production.

## Discussion

Cow manure is a common sustainable agriculture waste on factory farms. Cow manure left to decompose naturally could emit methane and nitrous oxide, two greenhouse gas emissions (GHGs) with potential damaging effect on the environment. Converting cow manure into a biofuel source and other chemicals might reduce GHGs and improve agricultural waste economics. The present study investigated the potential utilization of cow manure for lignocellulosic ethanol through pretreatment and enzymatic hydrolysis by the on-site method.

The main aim of the pretreatment process prior to enzymatic conversion was to disrupt the natural recalcitrance of the lignocellulose component in cow manure, which eventually increased ethanol production. We performed dilute-acid and alkali pretreatment methods to disrupt the structure of cow manure materials. Dilute-acid pretreatment hydrolyzed hemicellulose and resulted in a low content of xylose in solid residues. Alkali-pretreated cow manure could allow effective hydrolysis by lignocellulolytic enzymes and facilitate fermentation by recombinant *S. cerevisiae* LF1 converting five- and six carbon sugars into ethanol.

Cow manure used in the present study was composed of glucan (16.62%) and xylan (15.26%) by compositional analysis. Although the glucan contents remaining in the cow manure decreased compared with that of corn stover due to the degradation of lignocellulose faction through the cow’s digestive system, these agriculture wastes with relatively high carbohydrate content are still promising candidates of biodegradable sources due to their potential commercial application prospects in bioenergy.

Additionally, cow manure is a potential feedstock for the production of lignocellulosic ethanol not only because of its high lignocellulosic content but also because of its growing volume and convenient collection (Diaz et al. 2016). Compared with periodical corn stover collection, daily regular solid waste collection is more cost effective using an automated collecting machine of manure at its source. However, the distributed nature of corn stover has caused the substrate costs in ethanol production to average at $60 per ton (Johnson et al. 2016). Therefore, the easy and regular collection and abundant sources of cow manure could reduce the cost of bioethanol production and ultimately maintain high process stability and favorable conditions for rapid bioethanol production.

In general, an inducer is needed for efficient enzyme production. Cellulase expression was induced using corncob residues from xylose production to reduce medium cost. Alternatively, feeding of cow manure during cellulase production by *P. oxalicum* achieved high lignocellulosic enzyme productivities in the present study. The strategy might be also applicable to other cellulase-producing fungi (e.g., *T. reesei*). Collectively, shifting from off-site to on-site enzyme production was also an efficient strategy to reduce enzyme production costs. Then, constructing an energy-intensive route, a combined process from feedstock, milk, cow manure, lignocellulolytic enzyme, lignocellulosic ethanol, and biogas to fertilizer production, could be a sustainable option for a biofuel refinery, which might be economically feasible in factory farms.

**Fig. 5:**
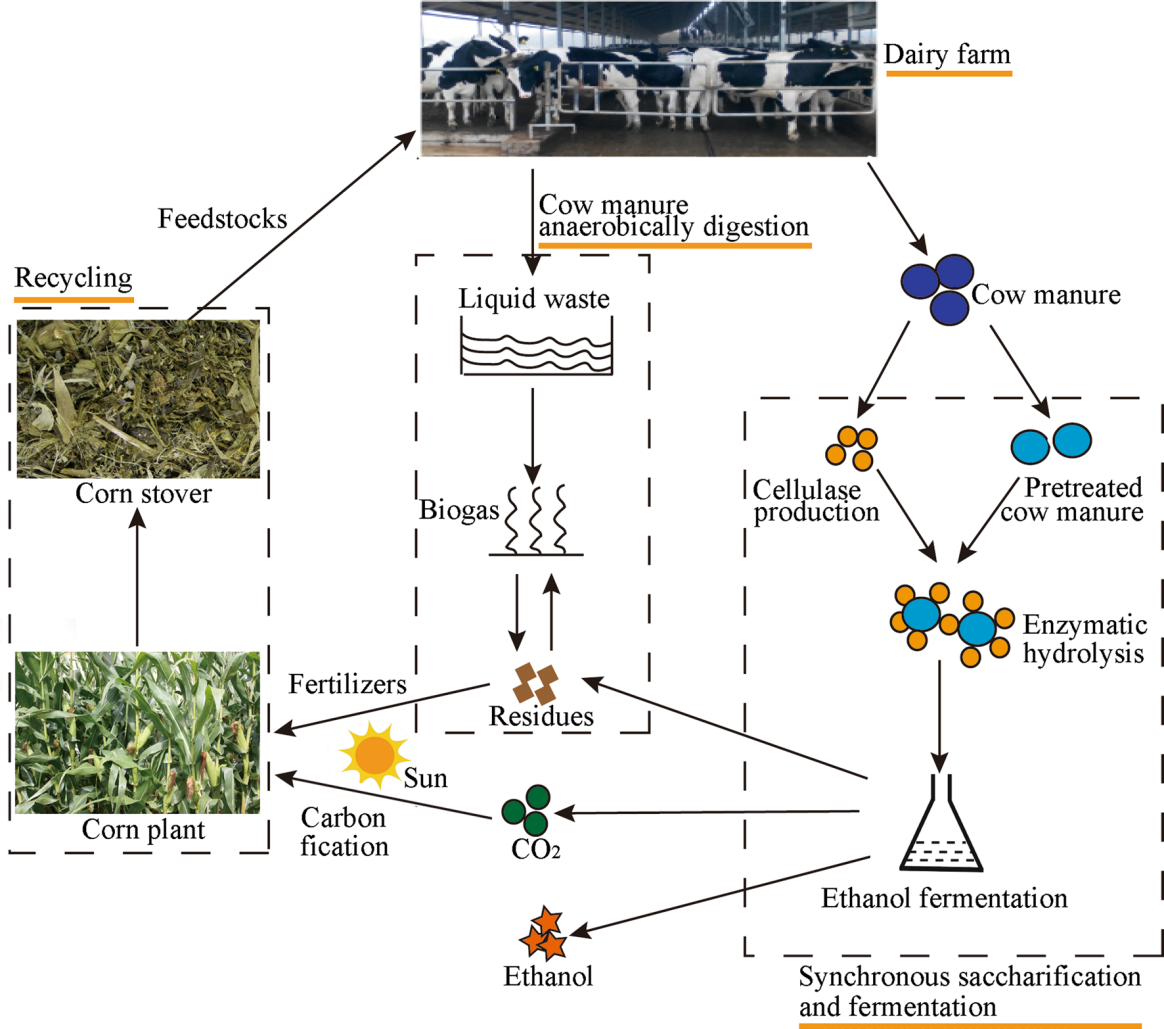
Integrated biorefinery approach using cow manure for cellulolytic enzyme, bioethanol and biogas production

The liquid part of the cow manure is first removed by mechanical packing to maintain the value of the cow manure. After the waste separation, the liquid cow manure fraction from manure materials might contain abundant organic substances (soluble starch, fiber, nitrogen, and phosphorus) (Diaz et al. 2016; Ma et al. 2017), which could be useful to produce biogas in anaerobic digesters (Fig. 5). The solid part of manure contains a small water content and a large carbohydrate content, which is subsequently subjected to pretreatment, enzymatic hydrolysis to yield glucose, and ethanol fermentation. The obtained residues from cow manure ethanol could be further mixed with the liquid cow manure fraction to improve the bio-methane potential via anaerobic fermentation (Fig. 5). The combined process for the co-production of bioethanol and biogas from cow manure might improve agricultural waste economics.

Laboratory trials of cow manure pretreatment and enzymatic hydrolysis revealed a strong possibility to produce bioethanol using readily available cow manure on factory farms. The integrated cellulase production using cow manure as the primary substrate by the on-site method can enhance bioethanol production and render lignocellulosic ethanol economically viable. Fed-batch enzymatic hydrolysis into fermentable sugars is a potential cow manure-bioethanol conversion method, but further extensive experimentation is warranted. From the biorefinery point of view, cow manure is a potential material for cellulosic ethanol production.

## Abbreviation

SSF: simultaneous saccharification and fermentation
